# Analysis and optimization of conventional furrow irrigation systems using the WinSRFR simulation model

**DOI:** 10.1038/s41598-026-48262-3

**Published:** 2026-07-13

**Authors:** Huma Zia, Fatima Shah, Nimra Imran, Nick R. Harris, Muhammad Khurram

**Affiliations:** 1 School of Physics, Engineering and Computer Science, University of Hertfordshire, AL10 9, Hatfield, United Kingdom; 2 Smart City Lab, National Center of Artificial Intelligence (NCAI), NED University of Engineering and Technology, Karachi, 75270 Pakistan; 3 Electronics and Computer Science, University of Southampton, Southampton, SO17 1BJ UK

**Keywords:** Energy science and technology, Hydrology

## Abstract

Surface irrigation systems continue to be the most popular irrigation systems worldwide, owing to their ability to save energy and ease of operation. They do, however, perform poorly as a result of the overall design and ineffective management. Therefore, the study aims to optimize the performance of conventional furrow irrigation systems in water constrained areas. Conventional furrow irrigation was used to collect field data and then to evaluate performance using the WinSRFR simulation model. The sensitivity analysis of WinSRFR revealed that the most sensitive parameters were inflow rate, cut-off time, and furrow spacing, respectively. The WinSRFR simulation model predicted that the conventional furrow design performed better in terms of application efficiency 84%, and achieved flow rates near the minimum allowable which is 0.0025m^3^/s in our case due to extreme water shortage. Therefore, given minimal runoff and percolation losses, with total maximum losses of around 16%, the best water application efficiency was achieved. Nevertheless, the lower quarter adequacy is 0.74, indicating that the field is under irrigated due to significant water constraints in the farm field. Simulation models of WinSRFR have proved to be an effective model for designing, forecasting, and optimizing the performance of conventional furrow irrigation systems. In earlier studies, researchers used fictitious scenarios to predict how well the furrow systems would perform by designing furrows with various lengths and flow rates on the same plot. The engineering approach used in this study was applied to actual crop fields, which greatly helped us predict the availability of the proper quantities of moisture to the crops and take the necessary action if the field was moisture deficient. Therefore, farmers can use our methodology to get deep insights into the performance of their fields and implement the necessary precautions to ensure the health of the crops.

## Introduction

The agriculture sector is the largest consumer of water in Pakistan, accounting for approximately 97% of the country’s total freshwater withdrawals, with the remaining percentage allocated to domestic and industrial uses^1^. Due to climatic and demographic changes, Pakistan’s water status has moved from “stressed” to “scarce”^2^. Optimizing irrigation systems to increase water uptake while decreasing water wastage is one step toward conserving water resources^3^.

Surface irrigation is the most common method of applying water to fields due to its low cost and energy consumption, as it relies on gravity rather than external energy sources like sprinkler and drip irrigation. Globally, surface irrigation accounts for 85% of all irrigation systems and more than 90% in Pakistan^4^. It is classified into three types: basin, border, and furrow irrigation. Furrow irrigation transports water across the field via small channels, passing through crop rows and providing adequate aeration to the root zone. Water infiltrates through the wetted perimeter, spreads vertically and horizontally to refill the soil reservoir, and avoids flooding the entire field as long as it flows down the channel^5^. Unlike border or basin irrigation systems, furrow irrigation allows independent control of water flow into each furrow. Traditional irrigation practices in the region typically involve manually regulated, non-engineered open-end furrows with no defined inflow rate, cut-off time, or standardized spacing. These systems, commonly referred to as conventional furrow irrigation, rely on farmer experience rather than hydraulic principles, resulting in low application efficiency (45–60%)with substantial losses occurring through deep percolation and tailwater runoff^6^. The performance of such systems is strongly influenced by controllable design variables–including furrow spacing, length, slope, inflow rate, and cut-off time, yet these parameters are generally overlooked or applied inconsistently by farmers^7,8^.

Various simulation models are now being used to determine optimal design parameters for furrow irrigation systems^9^. These models help increase yields, conserve water, and reduce farming inputs such as water and fertilizers while minimizing design time^10^. Surface irrigation systems can be simulated by mathematically describing the hydraulic properties of water as it moves across a field^11^. Commonly used models include the SIRMOD^12^, SURDEV^13^, WinSRFR^14^, SURCOS^15^, and SISCO models^16^. Among these, WinSRFR has consistently demonstrated higher accuracy in reproducing field-measured advance times, infiltrated depths, and runoff volumes^17^. It integrates event analysis, infiltration parameter estimation, hydraulic simulation, and operational optimization within a single framework. These capabilities make it a strong candidate for field-level optimization, particularly in water-limited environments.

Various studies have been carried out using different simulation models to evaluate the performance and precision of design alternatives to optimize the performance of surface irrigation systems. Furrow design and application parameters, climatic and atmospheric variables, and soil parameters are all set as inputs to the simulation models. Then, several parameter combinations are assessed to improve the furrow system’s efficiency. As shown in the numerous studies stated below, the flow rate, furrow length, cut-off time, number of furrows, and field slope are the most important factors that can be changed to improve the efficiency of the furrow system. Moreover, one of the most crucial soil factors in the development and assessment of surface irrigation techniques is infiltration^18^. In the case of furrow irrigation, the shape and size of the furrow affect the amount of water infiltrated because the wider the furrow, the greater the area of soil and water contact^19^. To evaluate a furrow system’s performance, performance parameters, including application efficiency, deep percolation losses, low quarter adequacy, and tailwater losses are analyzed.

A study comparing these models found that WinSRFR accurately simulated advance-recession times, infiltrated depth, and runoff volume, while SIRMOD also performed well. Results showed that surge irrigation improved application efficiency and distribution uniformity while reducing deep percolation and runoff. The study confirms that these models are valuable for designing efficient irrigation strategies in water-scarce regions, aligning with our research objective of optimizing furrow irrigation using WinSRFR^17^.

In a study that deals with the optimization of the furrow system by finding optimal values of flow rate, furrow length,, and cut-off time using FURDEV (a module of SURDEV) and AMALGAM in MATLAB. The main objectives were, decreasing the percolation and tail water ratios and increasing the water requirement efficiency. It used five open-ended furrow irrigations having, loam soil texture. The results showed that increasing the flow rate from 0 to 45 percent, furrow length from 23.2 to 66.9 percent, and cut-off time from 18.9 percent to 112 percent, will increase application efficiency from 13.8 to 44.9 percent. Efficiency in terms of water requirement increased as well, from 14.2 to 76.8 percent. It also caused a decrease in tail water ratio by 48.4-56.0%^20^.

Real time optimization of a furrow irrigated commercial cotton crop was done through the SISCO model in Australia via calculating the soil infiltration characteristics. Four irrigations on a group of 11 furrows (970m long and 1m apart) are observed. It used features like inflow rate, water advance towards the mid-point of the furrow length, and infiltration curve, and predicted the best time to cut off using a hydraulic simulation program. The output model made sensing, simulation, infiltration scaling, and optimization executed automatically. Application efficiency using optimization of the actual infiltration curve was found to be between 72 to 100% and the predicted time to cut-off was between 280 to 568min. Control hardware can be set up to apply the desired cut-off time^21^.

Two different furrow irrigated cotton crops from Australia and Pakistan were analyzed for optimization using the INFILT-V and SIRMOD models. Simulation of advance trajectories was done, using field length, infiltration characteristics, slope, target application depth, Manning coefficient of roughness n, and geometrical parameters of furrow and discharge as input, showing that they were passing through advance points chosen for infiltration. After simulation, infiltrated water distribution, water distribution uniformity, water application efficiency, advance-recession trajectory, runoff hydrograph, volume balance and other efficiencies were calculated. INFILT-V calculated cumulative infiltration and the SIRMOD model used five irrigation strategies. Total volume of water used was 40% less (963m3)in the optimized model, 72m3 for field P and 891m3 for field Q rather than usual farm management (1595m3) , 104m3 and 1491m3 for field P and Q respectively. However, a blueprint for this model can be made for achieving vital benefits^22^.

Modified WinSRFR is one of the most widely used tools for evaluating, simulating, and designing surface irrigation systems (basin, border, and furrow methods)^23^. Users can analyze the performance of irrigation events and estimate field-average infiltration parameters based on field measured data, formulate design and operational alternatives, and conduct simulation studies using an unsteady flow model using WinSRFR’s Event Analysis, Operation Analysis, Physical Design, and Simulation features. The WinSRFR development project has resulted in upgrades and modifications to current parameter estimation, design, and operations analysis techniques due to the necessity for functional integration. WinSRFR is primarily a practical tool, but it will also serve as a platform for future development of surface irrigation hydraulic modeling and analysis approaches.

The functionality of WinSRFR was defined by Bautista et al. (2009a)^23^ based on the analytical procedure generally used to examine and improve the hydraulic performance of surface irrigation systems. E.Bautista.et.al in their study analyzed a surface irrigation system using WinSRFR with an example application. Graded basin irrigation systems in Yuma-Mesa Irrigation and Drainage District (YMIDD), Yuma, Arizona have a test labeled GC/2-9-05, which was selected for the example. The event analysis tools of WinSRFR were used to evaluate the performance of irrigation systems and estimate their infiltration and hydraulic roughness properties along with Manning roughness coefficient n, which are needed for subsequent design and operations analyses. Infiltration functions were estimated using the post-irrigation volume balance (PIVB) method from field-measured geometry, inflow and outflow hydrographs, and advance and recession times. The Operations Analysis World is used to optimize the inflow rate Q and cutoff time tco. The study is carried out using performance contours, which show how selected performance indicators vary as a function of Q and tco. The application efficiency (AE), distribution uniformity of the minimum (DUmin), and runoff (RO), and deep percolation (DP) fractions are among the performance contour plots created by the software. The sensitivity of the optimized operational strategy, Q = 200 l/s and tco = 53 min was tested for deviations in infiltration properties.

Mazarei.et.al^24^ used theWinSRFR model in their study to improve furrow irrigation on field data from sugar cane farms of Iran. For the field study, nine blocked-ended furrows were used having 250 m length, 1.83 top width and 0.04 % slope with three cycles having inflow treatments of 1.0, 1.5 and 2.0 L/s and five inflow rates of 1.0, 1.5, 2.0, 2.5 and 3.0 L/s in the simulation (WinSRFR, Zero inertia model) study. The objective function (OF) having application efficiency, distribution uniformity, and deep percolation was also estimated for enhanced outcomes. In the field study, for the first cycle, the mean values of application efficiency, deep percolation, and distribution uniformity were 75.5, 24.5 and 74.0%. For the second cycle, 60.2, 39.8, and 76.6%, and for the third cycle 48.6, 51.4 and 74.6%, respectively. Finally, the high objective function is 52.6% (first irrigation), 42.5% (second irrigation), and 34.2% (third irrigation) with a recommended inflow rate of 1 L/s. It was also found that OF could be increased by 35.99% using 1.0 L/s inflow rate in comparison of 1.5 and 2.0 l/s. for furrow length from 250m to 200m, OF increased by 39.8% but decreased by 7.7% for 300m. When the slope varied from 0.04 to 0.03%, OF lowered by 0.9% but increased by 1% for a 0.05% slope. However, when irrigation parameters (inflow rate, cut-off time, field length) and their combinations were altered, OF improved by 25, 8.39, and 31%. In WinSRFR, for the field length 200m and slope 0.05%, an inflow rate of 3 L/s and a cut-off time of 379.5 min were proposed. Further using these parameters, the maximum application efficiency (77.6%), deep percolation (22.4%), distribution uniformity (77.9%), and objective function (43.9%) could be obtained.

Despite extensive simulation-based studies worldwide, most existing research relies on long furrow lengths (200–300 m), hypothetical scenarios, or well-supplied irrigation flows, which do not represent the realities of smallholder farms in arid regions like Pakistan. Moreover, earlier studies seldom combine engineering-based field characterization (soil infiltration testing, furrow profiling, soil-water availability classes) with real-field simulation and optimization. Prior work has also not addressed optimization under extremely constrained flow conditions, where potable water availability can limit discharge to as low as 0.0025 $\hbox {m}^{3}/\hbox {s}$–levels commonly faced by farmers in Karachi’s peri-urban agriculture.

To address these gaps, the present study evaluates and optimizes a conventional furrow irrigation system using WinSRFR under actual low-discharge conditions and short furrow lengths typical of smallholder fields. The research was conducted within an Agro Living Lab, a real-time experimental platform established in Gadap, Karachi, to support digital, data-driven farm management. This environment enabled the integration of soil engineering measurements, controlled field experiments, and simulation-based optimization. Detailed field data–such as soil texture via sedimentation analysis, multi-point infiltration using double-ring infiltrometers, furrow geometry profiling, and soil-water availability classes–were collected to ensure representative model inputs, addressing concerns raised in earlier studies regarding the lack of field calibration in irrigation simulations. This study focuses on optimizing the performance of conventional furrow irrigation systems in water-constrained areas using the WinSRFR simulation model. The goal is to identify the optimal combination of inflow rate, furrow spacing, cut-off time, and other factors such as furrow length, width, depth, and slope to improve irrigation efficiency and distribution uniformity. The objective is to minimize water losses due to runoff and percolation while maximizing application efficiency. The hypothesis driving this research is that optimizing inflow rate, cut-off time, and furrow spacing will significantly enhance water application efficiency and reduce overall water losses in conventional furrow irrigation systems. By adjusting these parameters based on field data and simulation outcomes, the optimized system is expected to outperform traditional irrigation practices in the region.

The knowledge gained from multiple cultivation cycles using data-driven engineering approaches will be compiled into a guidebook for growers, simplifying agricultural operations and decision-making. In February 2022, a 3.0-hectare study site was established in Gadap, Karachi, serving as the Agro Living Lab. This research environment integrates scientific inquiry with practical agricultural applications, enabling collaboration among researchers, agricultural experts, and local farmers to develop innovative, sustainable solutions. The site was selected due to its significant water concerns, prompting a study focused on optimizing surface (furrow) irrigation methods.

The novelty of this study lies in understanding soil and crop behavior during water application while considering water restrictions, such as a flow rate of only 0.0025 $\hbox {m}^{3}/\hbox {s}$ for 10 hours per day. The research investigates the plant root zone depth’s response to precise water application, ensuring crops receive the required moisture while optimizing furrow geometry. Additionally, it explores soil behavior using engineering techniques that are often overlooked by Pakistani farmers. The WinSRFR model, widely recognized for evaluating and designing surface irrigation systems, plays a key role in this study by analyzing furrow irrigation hydraulics. This approach aims to enhance application efficiency while minimizing runoff and deep percolation losses, accounting for soil, climate, and environmental constraints, particularly the availability of potable water. The findings and methodologies developed in this study can be replicated in other agricultural fields to optimize surface irrigation water use.

The novelty of this study lies in the following aspects:

- Field-based optimization under extremely limited inflow ($0.0025 \hbox {m}^{3}/\hbox {s}$), reflecting real-world water scarcity conditions not previously examined in Pakistan.
- Integration of engineering soil characterization with WinSRFR simulation, providing a comprehensive, data-driven optimization approach for conventional furrow systems.
- Application of WinSRFR on short (6 m) furrows, demonstrating its suitability for smallholder farm configurations where most global studies focus on much longer field lengths.
- Empirical evaluation of the plant root zone response under constrained water applications, validated with soil moisture profiling.
- Implementation within an operational Agro Living Lab, offering a replicable framework for real-field irrigation optimization in arid and semi-arid regions.

While this study primarily focuses on optimizing furrow irrigation using the WinSRFR model under specific field conditions, further research could explore theoretical and technological advancements to enhance the model’s predictive capabilities, particularly for sandy loam soils and arid regions.

## Methods

**Fig. 1:**
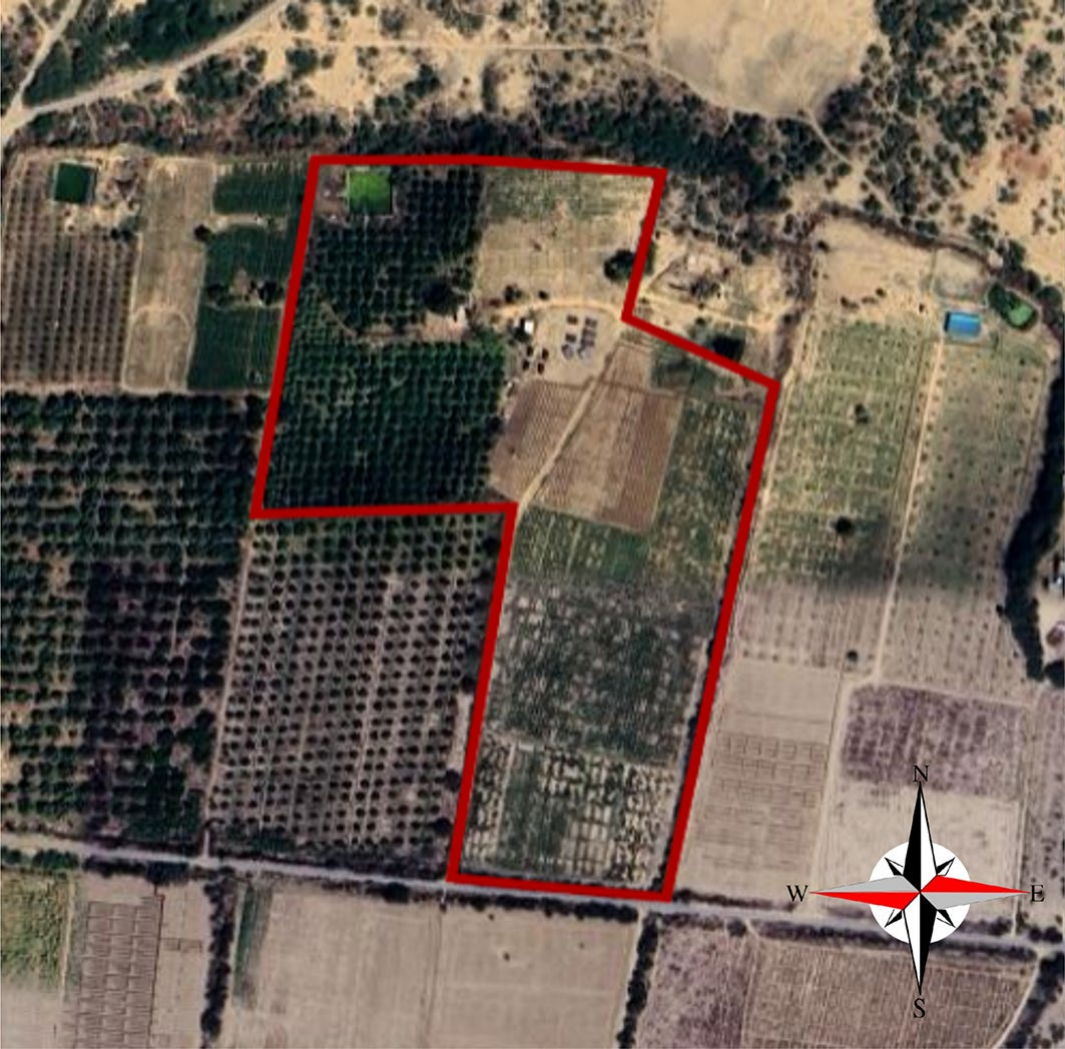
Study area at Gadap, Karachi.

Figure 1 shows the 3.0-hectare experimental farmland situated in the Gadap district of Karachi, Pakistan, located approximately between 25.147774$^{\circ }$N, 67.214164$^{\circ }$E, 25.147702$^{\circ }$N, 67.215846$^{\circ }$E, and 25.146698$^{\circ }$N, 67.215200$^{\circ }$E. The site functions as an Agro Living Lab, a real-field digital research environment for applying data-driven agricultural engineering methods. Due to chronic canal water shortages in the region, farmers rely heavily on tube-well water with limited discharge. This environment provides a realistic setting for analyzing and optimizing a conventional furrow irrigation system under water-scarce conditions.

The initial research activity within the Agro Living Lab focused on evaluating and optimizing furrow irrigation performance using the WinSRFR simulation tool–a widely validated hydraulic analysis model for surface irrigation that integrates evaluation, simulation, operational analysis, and design functionalities.

Intercropping of okra, bitter gourd, sponge gourd, and papaya was practiced on the site, reflecting typical smallholder farming practices in Gadap. The furrow system was designed and constructed using standard local methods, representing traditional irrigation practices–i.e., farmer-made furrows without engineering design, no optimization of inflow rate or cut-off time, and water application governed only by water availability rather than hydraulic criteria.

### Field testing and soil characterization

Accurate soil characterization is essential for surface irrigation modeling because field parameters cannot be selected or assumed–they must be measured. Therefore, a series of field tests were conducted to determine the soil texture, infiltration characteristics, soil moisture availability, furrow geometry, and field slope. These measured parameters formed the foundational inputs for developing the infiltration model, defining hydraulic behavior, and configuring the WinSRFR simulation for reliable evaluation and optimization of the furrow irrigation system.

#### Soil texture analysis

Sedimentation analysis (hydrometer method) was used to determine the soil texture for the study. This method was selected over alternatives such as sieve analysis or laser diffraction because it is the standard approach recommended for agricultural field soils and provides reliable separation of clay and silt fractions, which strongly influence infiltration behavior. It is also particularly suitable for sandy loam soils–the dominant texture in the Gadap region–and is widely employed in previous surface irrigation studies, ensuring consistency and comparability with existing research. The results confirmed that the soil at the study site is sandy loam, aligning with regional soil survey data.

#### Infiltration measurement using double-ring infiltrometer

Furrow irrigation generally exhibits bimodal infiltration behavior, with an initial rapid intake followed by a steady infiltration rate. Although methods such as block-furrow infiltration tests or infiltrometer strips can capture both vertical and lateral infiltration, these approaches were impractical at the study site due to the short furrow length and field constraints. Consequently, the double-ring infiltrometer was selected because it is recommended by the FAO for surface infiltration assessment, and allows lateral infiltration to be incorporated later in the WinSRFR model through adjustment of infiltration coefficients.

To capture soil infiltration variability along the furrows, we conducted the double-ring infiltrometer test at multiple locations. The following steps were taken:

- Setup: Two concentric metal rings (with inner and outer diameters of approximately 30 cm and 60 cm, respectively) were inserted about 5 cm into the furrow soil. This setup minimizes lateral flow, ensuring that the measurement focuses on vertical infiltration.
- Water Application: Water was simultaneously added to both rings, maintaining a consistent head in each. This method promotes vertical water movement in the inner ring, allowing accurate measurement of surface infiltration without interference from lateral flow.
- Data Collection: The rate of water infiltration through the soil surface in the inner ring was recorded over time, producing reliable infiltration data for model input.

#### Accounting for field variability

To account for field variability, we repeated the infiltration test at four separate points along the furrow. Testing at multiple locations ensured that our calculated infiltration rate reflected overall field conditions rather than isolated measurements. This comprehensive approach enabled the WinSRFR model to integrate natural variations in soil characteristics across the field, providing a more accurate simulation.

#### Consideration of furrow compaction

Tests were conducted on freshly prepared furrows at the start of the irrigation season when the soil was relatively loose. Recognizing that soil compaction would likely reduce infiltration over time, we incorporated a variable infiltration rate parameter in the WinSRFR model to reflect the expected changes throughout the season. This adjustment allowed the model to simulate more realistic water movement dynamics across different stages of the season, thus improving the reliability of the predictions.

#### Application of the modified kostiakov equation

For this study, we applied the Modified Kostiakov equation to model the infiltration behavior of sandy loam soil, as it accommodates the transition to a steady state infiltration rate over time unlike the original Kostiakov equation, which assumes a continuously declining rate. The Modified Kostiakov equation is particularly suitable for furrow irrigation as it introduces a long term infiltration rate parameter (b) that more accurately reflects real-world conditions in surface irrigation. To determine a suitable value for b, we observed the steady state infiltration rate after 7 hours, noting a consistent infiltration rate of 6 mm/hour. In this application, the adjustment constant c was set to zero, assuming no further corrections were necessary. We selected an exponent a=0.5, a typical value for sandy loam soils that captures the decline in infiltration over time^25^. The initial infiltration constant k was set to 7 mm/hour, based on field data. These values were input into the WinSRFR model, allowing for an accurate simulation of soil water interactions and providing insights into effective irrigation practices. Table 1 shows a flow diagram of the steps taken to conduct the study.

The Modified Kostiakov equation, which adjusts for a steady state infiltration rate, can be expressed as:

1 $$\begin{aligned} z = k \cdot \tau ^a + b \cdot \tau + c \end{aligned}$$

In this equation: - $z$ represents the cumulative infiltration (L), - $\tau$ is the opportunity time (T), - $k$ is the infiltration rate constant (L/T$^a$), - $a$ is the dimensionless exponent (where $0< a < 1$), - $b$ is the steady-state infiltration rate (L/T), and - $c$ is an instantaneous infiltration volume representing macropore infiltration (L)^25^.

**Table 1:** Flowchart steps for surface irrigation simulation.

Step	Description
1	Field data collection: Various soil tests, conventional furrow geometry, type of crops to be cultivated, root zone water requirements.
2	Use WinSRFR, a one-dimensional simulating tool for hydraulic analysis of surface irrigation systems.
3	Select Furrow as input and provide required parameters: depth, system geometry (parabola), topography (slope), width (100 mm), top width, maximum furrow depth (Ymax), base, spacing, and length of furrow (6m).
4	Input Infiltration Function (Advanced Kostiakov Formula), Manning’s roughness coefficient (n), and soil-crop parameters.
5	Input Inflow/Runoff parameters: inflow rate, cutoff options, and downstream conditions.
6	Choose the Solution Model (Kinematic-Wave).
7	Analyze Simulation Results: Advance time, recession time, infiltrated depth along the furrow, irrigation efficiency, distribution uniformity, deep percolation losses, and runoff loss.

### Soil water availability classes

Soil water availability classes were determined at various depths ranging from 0 to 60 cm with 20 cm intervals. For that, a small piece of farmland was irrigated and left for 24 hours to gain better insights into available water at the specified depth, where the root zone depth of various crops is expected to reach throughout their entire life cycle. The soil classes are Field Capacity (FC), Wilting Point (WP), and Available Water (AW), which were determined using a soil probe and then validated with Richard H. Cuenca’s irrigation system design manual^25^. The moisture availability at various depths assisted in predicting the depth of infiltrated water required to replace soil water deficits at the end of an irrigation event, as well as the frequency of irrigation. Table 2 lists the moisture values for each class.

**Table 2:** Tabulated moisture values for each class.

Soil depth (cm)	FC (%)	WP (%)	AW (%)	Texture
0 - 20	19.7	6.4	13.3	SL
20 - 40	21.3	8.0	13.3	SL
40 - 60	22.0	9.2	12.8	SL

### Field data

For the optimization of a furrow irrigation system using a simulation model of WinSRFF 5.1.1, field data were collected as input and calibration of the simulation model, which includes, field slope(m/m), maximum infiltration depth (mm), furrow geometry i.e. its shape, top and bottom width (mm), depth (mm), side slope (m/m). The length of furrow and number of furrows per set were selected based on the type of crops to be cultivated, soil type, and the inflow rate. The Manning’s roughness coefficient was selected based on barren conditions. The input parameters are tabulated in Table 3, and the reasons behind the selection of their value are described in the following paragraphs. Whereas, application efficiency (%), low-quarter adequacy (%), tail water ratio (%), and deep percolation loss (%) are predicted using the simulation model of WinSRFR 5.1.1.

2 $$\begin{aligned} \eta _a = \frac{W_z}{W_l} \times 100 \end{aligned}$$

where:

- $\eta _a$: Water application efficiency (%),
- $W_z$: Water stored in the plant root zone,
- $W_l$: Water applied to the land.

### Procedure for assessing conventional furrow irrigation system

When evaluating a surface irrigation system, different management practices that can be implemented to improve irrigation system efficiency are identified^26^. These practices may include a reduction in flow rate and application time, changes in field length, or a combination of diverse strategies. The primary objective of the WinSRFR software is to aid in exploring surface irrigation management strategies that result in satisfactory efficiency levels. To evaluate conventional furrow irrigation methods, the following section presents a comprehensive description of the data gathered from the field through engineered practices, as outlined in Table 2.

**Table 3:** Input parameters collected from the field.

Parameter	Measured value
Soil type	Sandy loam (SL)
Furrow geometry	Power law
Top width	600 mm
Width at 100mm depth	305 mm
Base	200 mm
Maximum depth	300 mm
Field slope	0.50%
Required Infiltration depth	100 mm
Length of furrow	6 m
No. of furrows	90
Manning’s roughness coefficient (n)	0.04
Inflow rate	0.0025 m^3^/s
Cut-off time	8 hr
Downstream condition	Open end

####  Field slope

Field slopes are influenced to some extent by the natural grade of the land to be irrigated^27^. In this study, furrow lengths were kept short, and a 0.5% grade was chosen, as recommended in Richard H. Cuenca’s design manual of surface irrigation systems, which states that grades up to 0.5 percent may be acceptable in areas if run lengths are kept short enough that water does not accumulate to the point where it causes erosion^25^.

#### Furrow cross-section

**Fig. 2:**
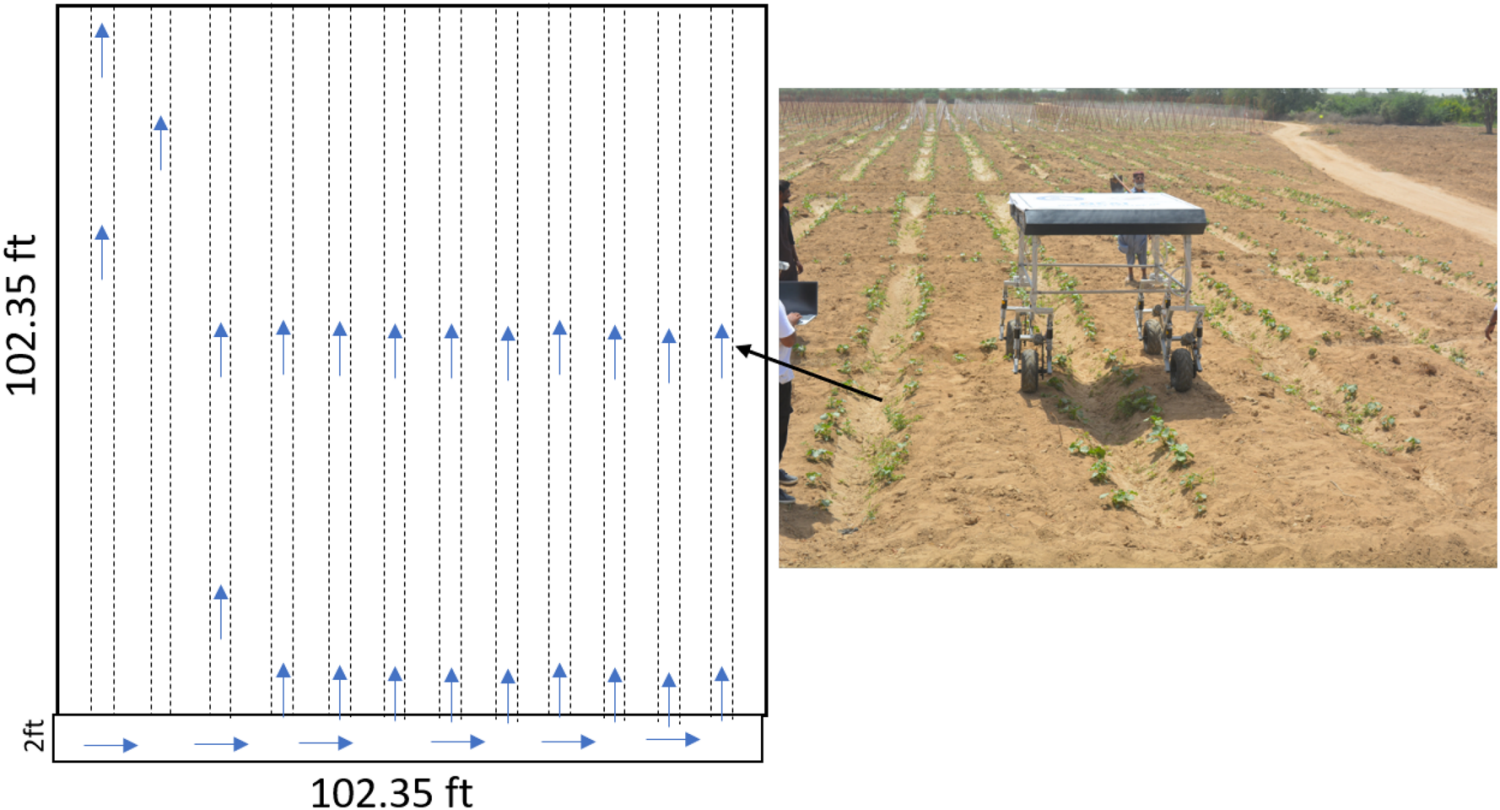
Layout of furrow irrigation system.

**Fig. 3:**
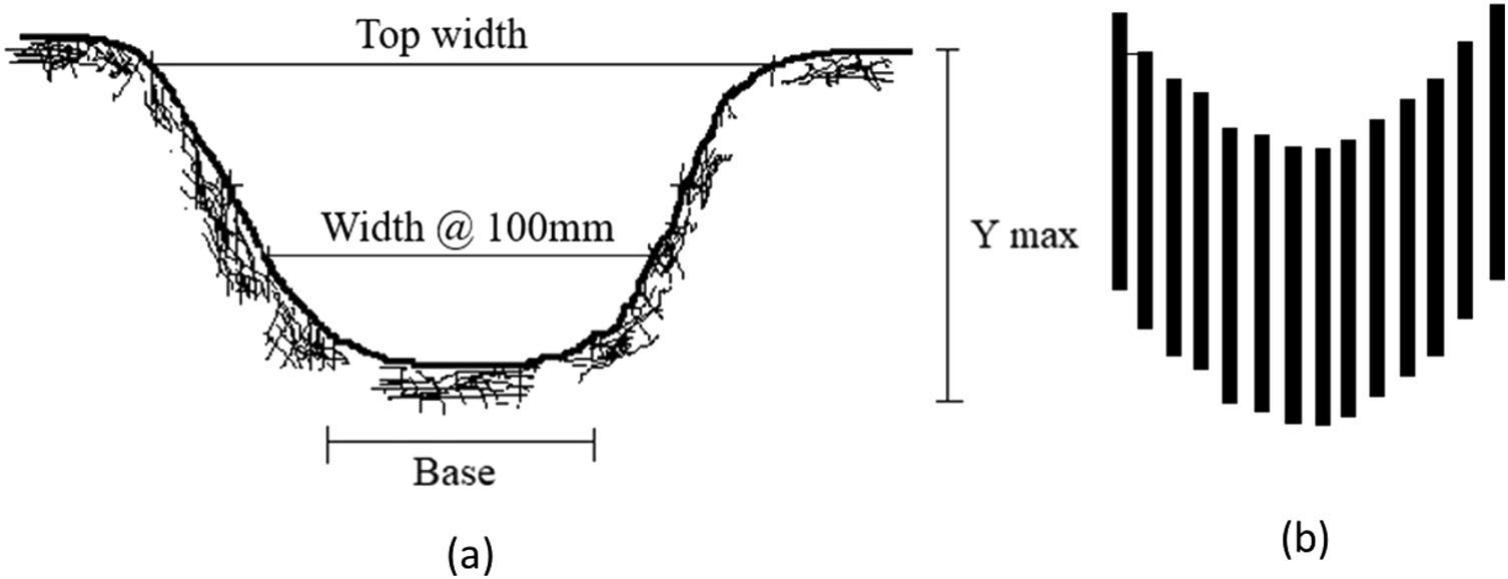
(**a**) Furrow cross-section. (**b**) Pegs at 50 mm spacing used to collected infiltrometer data.

**Fig. 4:**
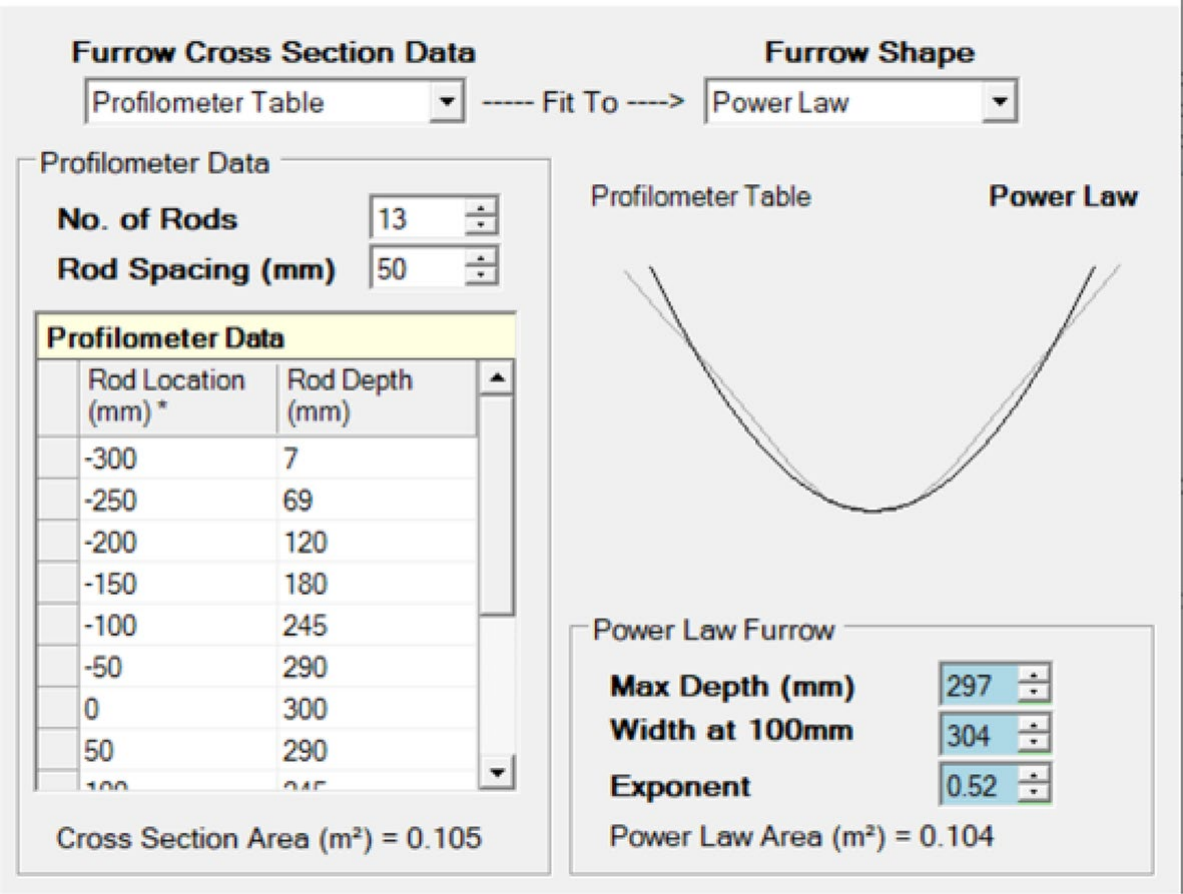
WinSRFR cross-section window, displaying the furrow cross-section based on inputted profilometer data.

The lengths of the furrow system were decided by dividing a 0.40 hectare farm field into 4 equal plots; an illustration of the division and design of the furrow system on one of the four plots can be depicted in Fig. 2. The furrows were designed with a parabolic shape. A profilometer is used to measure the distance between an arbitrary plane above the furrow and the furrow itself. In an X-Y coordinate system, the measurement is taken at multiple points along the furrow width, with Y values representing rod depth and intervals between values representing rod spacing. Profilometer data analysis is based on an approximately symmetrical geometry, a coordinate system with the origin in the middle (X) and top (Y) of the furrow, and constant rod spacing. As a result, X values range from negative to positive, whereas Y values are always positive. Three separate flow sections were considered and the resulting flow areas were then fit as a function of depth to a parabola to develop an approximate geometry. Table x shows the average width and depth values.A parabolic cross-section of the furrow and the arrangement of pegs for collecting infiltrometer data to facilitate smooth cross-section design are depicted in Fig. 3. Table 4 presents field profilometer data, while Fig. 4 displays a WinSRFR furrow cross-section window, where field profilometer data was inputted to verify and adjust the cross-section shape in line with software recommendations. The alignment between the field values and WinSRFR’s suggested parabolic shape was satisfactory.

**Table 4:** Profilometer data.

X (mm) (Distance)	Y (mm) (Depth)
300	7
250	69
200	120
150	180
100	245
50	290
0	300
-50	290
-100	245
-150	180
-200	120
-250	69
-300	7

####  Fertigation technique

Fertigation was applied using organic manure as a solute carrier in the irrigation water, following traditional agronomic practice. Water from a Bedford storage pond and wells was conveyed through pipes and open furrows to irrigate the crops. No chemical fertilizers or precise dosing equipment were used, and fertilizer concentration was not measured, as the focus was on crop irrigation and hydraulic performance rather than nutrient flow.

#### Cut-off time $T_{c_o}$

The time of cut-off is the beginning of the depletion phase. The depletion phase continues until the soil surface at the head of the field is again visible and is no longer submerged. For the area under study cutoff time, depends upon the availability of water, and its flow rates. The average cut-off time of the farmland was 8 hours.

#### Optimal flow

The optimal flow rate was calculated on the field using the bucket method to determine the relationship between flow rate and water application efficiency, percolation, and runoff losses. The time it takes to fill a bucket of known volume is used to calculate the flow rate. The entire flow is diverted into a suitable sized bucket, and the time it takes to fill it is timed using a stopwatch. The flow rate is calculated by dividing the bucket volume by the filling time. To obtain more accurate results, the procedure was repeated five to seven times to get an average filling time. The results were measured in cubic meter/second This method is appropriate for measuring flow rates in small streams or channels where the entire flow can be diverted to a bucket. The bucket should not be filled by holding it below the water’s surface, as this does not provide accurate flow rate values. The method’s limitations for measuring only small flows, approximately 5-6 l/s, stem from the need to divert all of the flow into the bucket, which may be difficult for streams with high flow rates. Secondly, accuracy can suffer if the filling happens too quickly, especially if it only takes a few seconds. The variations in filling times between separate measurements provide a good indication of the results’ accuracy. After taking multiple values in the field an average of 0.0025m^3^/s was calculated. To accurately represent flow rate variability in our modeling, we utilized the capabilities of the WinSRFR software. The WinSRFR model allows the flow rate to be input as a fixed nominal value or adjusted using the Cutoff time Options, which can simulate inflow rate variations over time. By applying these options in our model setup, we ensured that the simulation captured the slight but realistic fluctuations in flow rate observed during field measurements. This approach allowed our model to better reflect the dynamic conditions of the field, providing a more comprehensive and accurate analysis of irrigation performance.

####  Kinematic wave model

This study employs the kinematic wave model, which is a simplified version of the hydrodynamic model. It is assumed that there is no height variation of flux with distance and that the force due to the weight component in the direction of flow is balanced with friction forces, that is, $\frac{\partial y}{\partial x} = 0$, ignoring the momentum equation entirely, leaving the continuity equation ( equation 1) undetermined in term $\frac{\partial A}{\partial t}$^25^. To solve this problem, the momentum equation is replaced by the Manning equation (equation 2), assuming that there is a unique relationship that describes flow as a function of flow area. As a result, the equations that comprise the kinematic wave model (Miller, 1984) are transformed into continuity equations:

3 $$\begin{aligned} & \frac{\partial A}{\partial t} + \frac{\partial Q}{\partial x} + \frac{\partial Z}{\partial \tau } = 0 \end{aligned}$$

4 $$\begin{aligned} & A = \sqrt{\frac{Q^2 \cdot n^2}{S_0 \cdot R^{4/3}} } \end{aligned}$$

- *A* = Cross-sectional flow area ($\hbox {m}^{2}$)
- *Q* = Flow discharge ($$\hbox {m}^{3}/\hbox {s}$$)
- *x* = Distance along the flow path (m)
- *t* = Time (s)
- *Z* = Water surface elevation (m)
- $\tau$ = Time variable associated with elevation changes
- *n* = Manning’s roughness coefficient (dimensionless)
- $S_0$ = Channel bed slope (dimensionless)
- *R* = Hydraulic radius (m)

## Results

The results section offers a concise interpretation of the graphs generated through the WinSRFR simulation model. The primary aim of these results is to understand the behavior of the plant root zone depth under minimal water supply conditions facilitated by conventional furrow geometries. Additionally, the study aimed to enhance the understanding of soil behavior by implementing diverse engineering approaches, addressing the gaps prevalent in the adoption of these techniques within the Pakistani farming community. Utilizing the WinSRFR model, a widely acclaimed tool for evaluating, simulating, and designing surface irrigation systems, this research delves into the hydraulics of furrow irrigation. The model’s application aims to boost efficiency in water application, simultaneously minimizing runoff and deep percolation losses. This analysis takes into account various factors, including soil characteristics, climatic conditions, and environmental constraints, notably the availability of potable water.

**Fig. 5:**
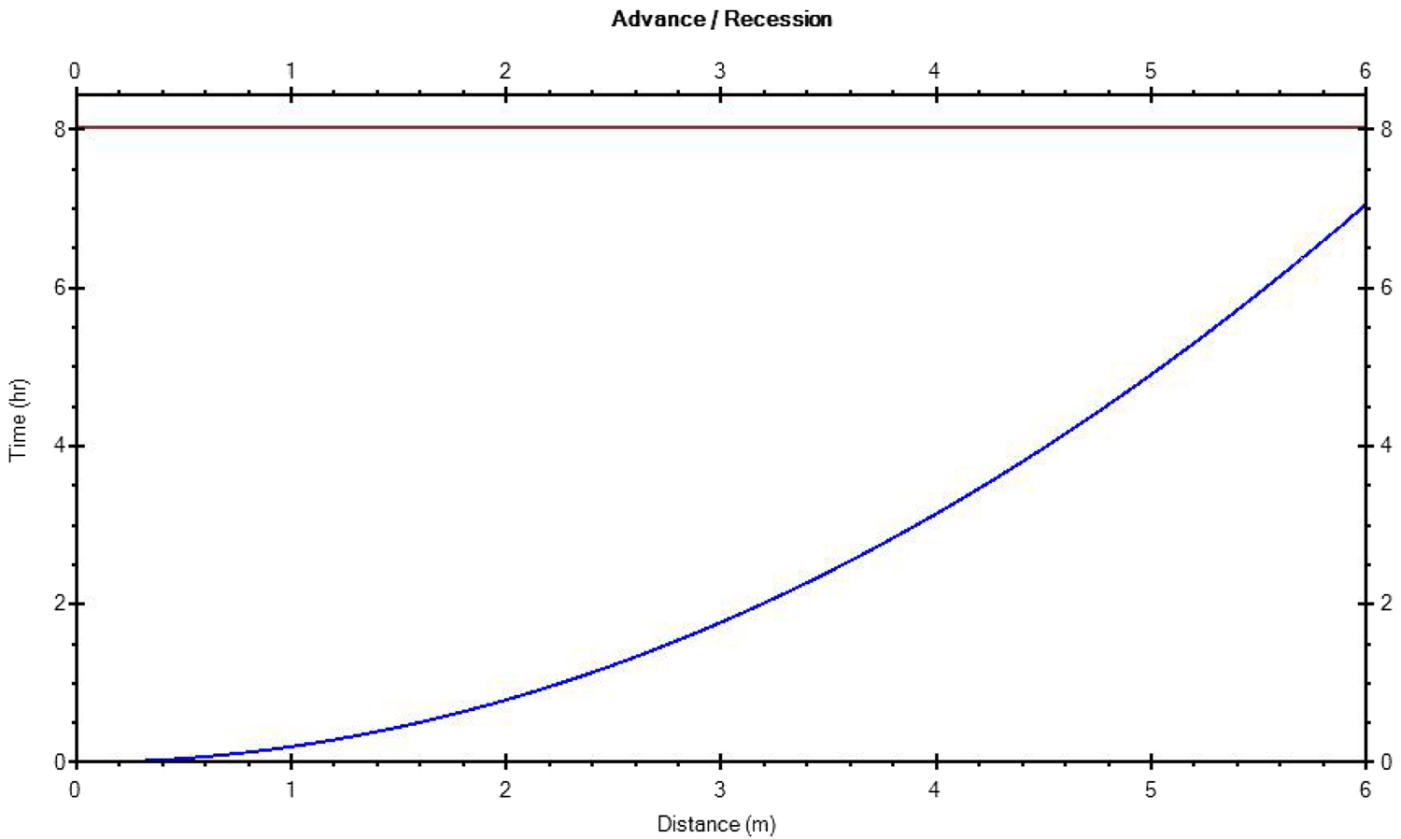
Advance and recession times, mapping irrigation efficiency through cutoff time alignment with furrow length and water movement dynamics.

The meticulous examination of the advance/recession curve provides valuable insights into the dynamics of water distribution and soil moisture management in the field. In Fig. 5, the Y-axis, representing the cutoff time of 8 hours, is intricately linked to the 6-meter length of a single furrow. The blue line represents the advance curve, which shows the movement of water along the furrow over time. It illustrates how the water front progresses from the inlet to the end of the furrow.

The red line represents the recession curve, which indicates when the water starts to recede from different points along the furrow after the inflow is stopped. The 8-hour cutoff time reflects the unique characteristics of the study site, including high infiltration rates and low water availability. This duration ensures optimal soil wetting and minimal losses, as validated through field measurements and soil moisture checks. This length accommodates 90 furrows within the 108-meter width of the plot, with 16 furrow beds along the 108-meter length. As the model charts the advance and recession curves against the backdrop of a single furrow’s length, it becomes evident that the irrigation system’s performance is closely tied to the efficient movement of water down the field.

During the advanced phase, depicted by the blue curve, water moves across the field, and the model captures data by measuring the time required for water to reach varying distances. The subsequent recession phase, illustrated by the nearly straight brown line, is notable for its negligible extent. This minimal recession is attributed to the limited water availability for irrigation, strategically meeting crop requirements and ensuring thorough soil wetting. The precision of this approach is further validated through on-site excavations and soil moisture checks, with soil probes indicating a field capacity ranging from 20% to 22%.

The vertical distance between the advance and recession curves, representing the infiltration opportunity time, emerges as a pivotal metric. This metric serves as a comprehensive assessment of irrigation effectiveness, allowing stakeholders to gauge whether the irrigation water has reached the optimal depth of the crop root zone. The observed minimal recession underscores the accuracy of the irrigation model, affirming its ability to achieve thorough soil wetting, facilitate optimal root development, and strategically utilize water resources where they yield maximum benefit for the crops. This connection between the advance/recession curve analysis and the irrigation system’s precision highlights a holistic approach to water management, integrating modeling, field observations, and soil moisture checks to ensure sustainable and efficient agricultural practices.

**Fig. 6:**
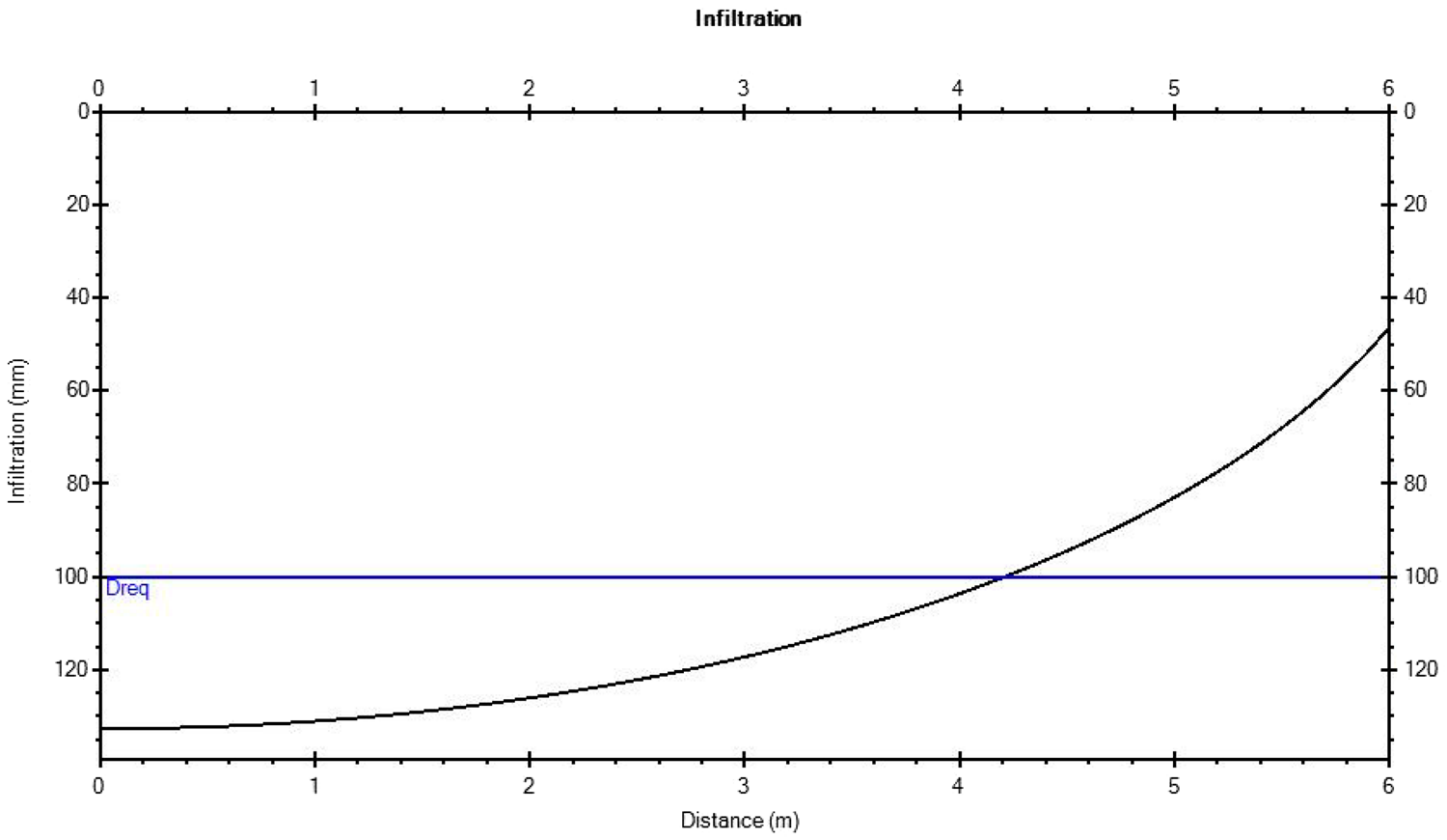
Graph showing the infiltration depth across the furrow length, generated using the WinSRFR simulation model.

The interpretation of the infiltration depth graph, generated through the WinSRFR simulation model, provides valuable insights into the behavior of sandy loam soil under irrigation. The infiltration rate, determined to be 7mm/hr through infiltrometer testing, is depicted graphically in Fig. 6. A blue line on the graph represents the infiltration depth, set at 100mm, which is considered an optimal depth for crop root zones to prevent water deficit conditions. Given the sandy loam nature of the soil, the irrigation water efficiently infiltrates at the initiation of the furrow. The graph illustrates this initial rapid infiltration, reflecting the soil’s propensity to absorb water readily. However, as the stream of water progresses along the length of the furrow, there is a noticeable reduction in losses. The represented blue line, indicating the optimal depth for crop root zones, aligns with the maintenance of water within the root zone to avoid deficit conditions. Notably, the simulation model reveals that only 14% of the infiltrated water experiences deep percolation losses. This implies that the irrigation strategy is effective in minimizing wastage and promoting water retention within the root zone, a critical factor for avoiding water deficit conditions in crop development. The interpretation underscores the significance of understanding soil-specific characteristics and employing tailored irrigation techniques to optimize water use, mitigate losses, and ensure favorable conditions for crop growth in sandy loam soils.

**Fig. 7:**
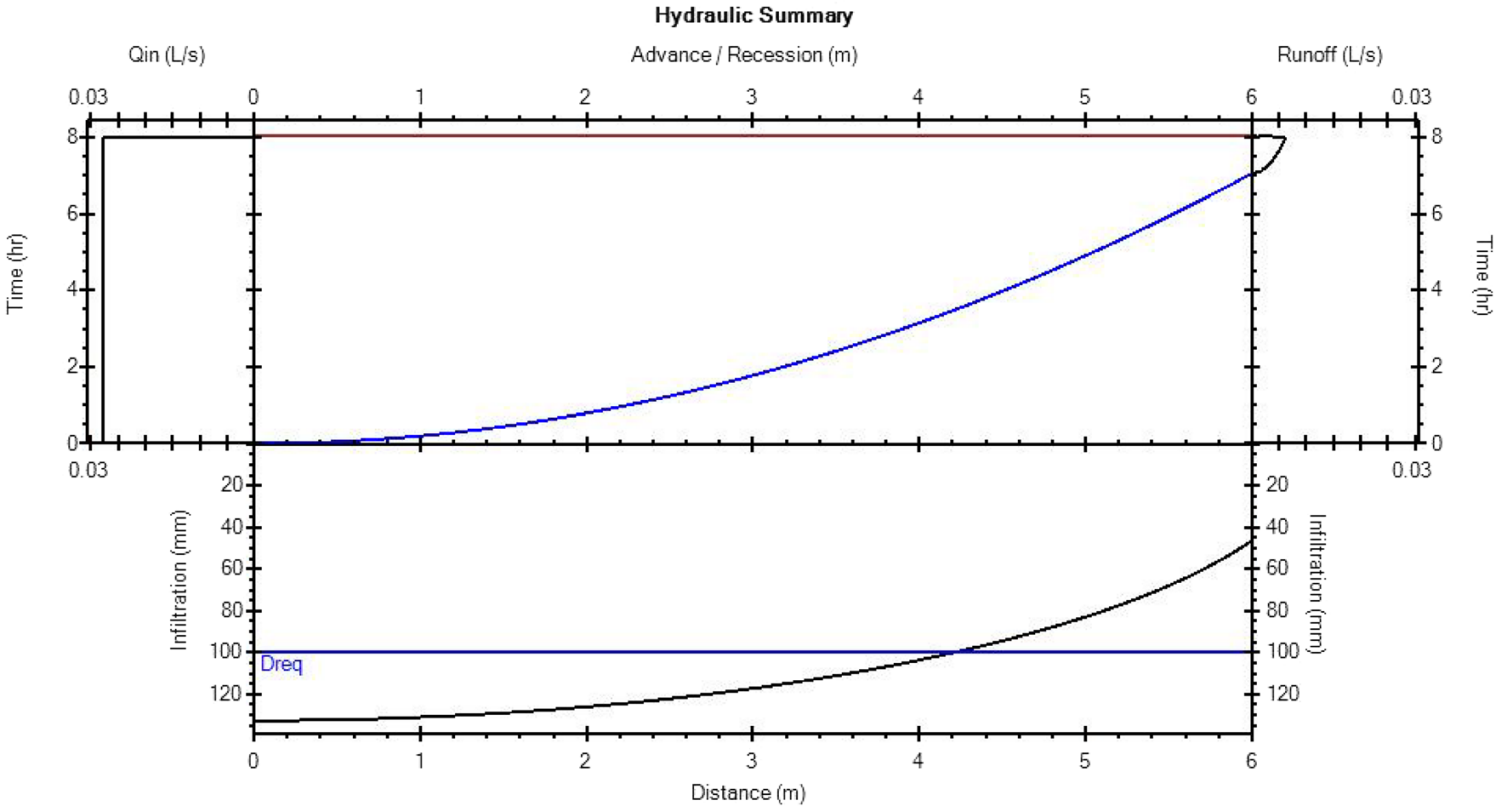
A hydraulic overview of the furrow irrigation system, providing a comprehensive examination of important factors like recession and advance curves, deep percolation, and runoff losses.

**Fig. 8:**
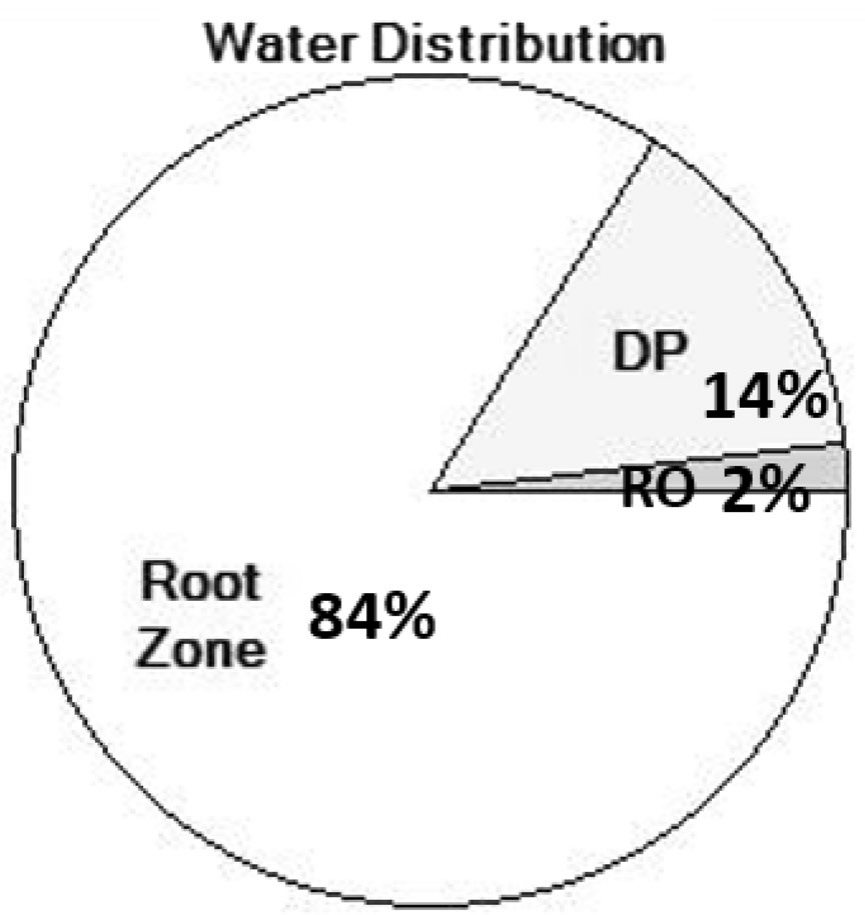
The allocation of irrigation water depicted in a pie chart, demonstrating a noteworthy water application efficiency of 84%, with 14% attributed to runoff (RO) and 16% to deep percolation (DP) losses.

Figure 7 presents a comprehensive hydraulic summary of the furrow irrigation system, offering a detailed overview of key parameters such as recession and advance curves, deep percolation, and runoff losses. This graphical representation serves as a valuable tool to gain profound insights into the dynamic movement of irrigation water over time across the plot. The hydraulic summary delineates the temporal evolution of water distribution and highlights distinct phases that characterize the entire irrigation process. The graphical representation allows for the segmentation of water movement into various phases, each contributing to the overall understanding of the system’s behavior. By examining the recession and advance curves, observers can discern the temporal dynamics of water as it moves along the plot during different stages of the irrigation cycle. These curves provide crucial information about the timing and efficiency of water distribution. Deep percolation and runoff losses, depicted in Fig. 8, offer additional layers of insight. These aspects quantify the extent of water that penetrates beyond the root zone (deep percolation) and the portion that runs off the field surface. Understanding these losses is instrumental in evaluating the overall efficiency of the irrigation system and identifying opportunities for optimization.

Figure 8 illustrates the distribution of irrigation water through a pie chart, showcasing a notable water application efficiency of 84%. This achievement is attributed to a substantial reduction in both deep percolation and runoff losses. In comparison to conventional furrow irrigation systems, where application efficiency typically hovers around 65%, the design implemented here has surpassed expectations, attaining more than satisfactory results. The pie chart visually communicates how the majority of the irrigation water is effectively utilized for crop nourishment, with only a minimal proportion lost through deep percolation and runoff. This high water application efficiency, a testament to the careful design and implementation, reflects an improvement over traditional practices. Conventional furrow irrigation systems have struggled to achieve efficiencies beyond 65%, making this advanced system a notable success in optimizing water utilization.

This outcome emphasizes the efficacy of a vigilant approach, coupled with the application of advanced models and engineering techniques. Even in situations with limited water availability, the use of innovative irrigation methods allows for a significant enhancement in the efficiency of conventional furrow irrigation systems. This not only conserves water but also promotes the optimal growth of crops, showcasing the potential for sustainable and effective irrigation practices in agriculture.

## Discussion

The Discussion should be succinct and must not contain subheadings. This study aimed to understand the behavior of soil and crops during water application under conditions of limited water availability, specifically focusing on how to optimize water distribution in furrow irrigation systems. The primary goal was to ensure that the plant root zone receives adequate water for crop nourishment, while accounting for the constraints imposed by water restrictions. In this context, the study also aimed to address the lack of engineering approaches in the Pakistani farming community by introducing a more systematic method for designing and optimizing the geometry of conventional furrows.

A detailed study was conducted in Gadap, Karachi, using the WinSRFR simulation model to assess the performance of a conventional furrow irrigation system under sandy loam conditions. Through this research, we explored key soil properties and irrigation parameters that directly influence irrigation performance, such as inflow rate, cut-off time, and furrow spacing. The sensitivity analysis conducted within the WinSRFR model highlighted that these parameters were the most influential in optimizing irrigation performance.

The sensitivity analysis revealed that the applied flow rate was a significant factor in system performance, with the best results achieved at near-minimum allowable flow rates. In this study, the field was located in a water-scarce area, and a flow rate of 0.0025 $\hbox {m}^{3}/\hbox {s}$ was used. This minimal water application rate led to high water application efficiency with minimal losses. Adjusting furrow spacing was also crucial, spacing set at 0.3 m resulted in the highest efficiency, and any increase above this value caused a marked decrease in efficiency. Furthermore, the cut-off time was optimized at 8 hours, ensuring that the field received full irrigation. Any deviation from this duration resulted in a significant reduction in water application efficiency.

In terms of optimization, the study found that the length and slope of the furrows had little effect, especially in soils with high infiltration rates, which could achieve optimization across a wide range of values for these parameters. One of the most significant challenges identified was minimizing percolation losses, particularly in sandy soils with high infiltration rates. However, in soils with low infiltration rates, both percolation and runoff losses could be easily minimized, contributing to better system efficiency.

The WinSRFR model predicted that the conventional furrow irrigation design performed with an application efficiency of 84%, which was considered high compared to the typical efficiency of around 65%. This efficiency was achieved by using flow rates near the minimum allowable levels, thus minimizing both runoff and percolation losses. However, it is important to note that while the application efficiency was high, the lower quarter adequacy was 0.74, indicating that the field was still under-irrigated due to water shortages.

This finding underscores the importance of maximizing distribution uniformity and application efficiency in furrow irrigation. By carefully selecting and optimizing key field parameters, such as inflow rate, cut-off time, and furrow spacing, it is possible to achieve more efficient irrigation systems, even under severe water scarcity. The engineering approach used in this study provided an effective tool for predicting moisture availability in the soil, allowing for timely interventions to prevent moisture deficiencies in the field.

While the study provides valuable insights into optimizing conventional furrow irrigation under water-limited conditions, further validation is needed to ensure the generalizability of the results. Future research should focus on validating the high application efficiency of 84% in other locations and under varying soil types. Additionally, investigating the long-term sustainability of such systems, including the impacts of soil compaction and variable weather conditions, will be essential to fully understand the practical application of these findings.

The parameters used in the WinSRFR model were derived from field measurements (e.g., infiltration rates, furrow geometry) rather than formal calibration due to data constraints. While this approach ensured a practical evaluation, future research should focus on parameter calibration and uncertainty quantification to enhance reliability and generalizability. Moreover, further exploration of techniques to reduce percolation losses in high-infiltration soils, such as the use of soil amendments or modifying furrow design, would help refine and improve irrigation efficiency. Continued research into automated irrigation systems, such as the integration of sensors for real-time monitoring and adjustments, could further enhance the precision and effectiveness of furrow irrigation in regions with limited water resources.

While this study primarily focuses on the application of the WinSRFR model for optimizing furrow irrigation under specific field conditions, further research could explore theoretical and technological advancements to enhance the model’s predictive capabilities, particularly for sandy loam soils and arid regions.

In conclusion, this study demonstrates that conventional furrow irrigation systems, when optimized with the right parameters, can achieve high water application efficiency, even in water-scarce areas. By providing a systematic approach to optimizing furrow irrigation geometry and accounting for soil properties, this research offers valuable guidance for improving irrigation practices in water-constrained regions, particularly for the farming communities in Pakistan and similar arid areas. Further validation and innovation in irrigation management will continue to be crucial for ensuring food security and sustainable water use in agriculture.

## Data Availability

All relevant data generated and analyzed during this study are included within the manuscript. This encompasses soil characterization details, infiltration rates, field testing procedures, soil water availability classifications, and parameter values used for the WinSRFR model simulations.;
